# Mental Fatigue Impairs Temporal Perceptual Prediction: A Study on Boxing Performance Across Skill Levels

**DOI:** 10.3390/sports13050154

**Published:** 2025-05-20

**Authors:** Chang-Hong Wu, Yi Yang, Xia Xu, Ning Wang, Qiao Li, Lu Geng, Shan-Jun Bao

**Affiliations:** 1 School of Sports Medicine, Wuhan Sports University, Wuhan 430079, China; 2022410170@whsu.edu.cn (N.W.); 2022410168@whsu.edu.cn (Q.L.); 2021420010@whsu.edu.cn (L.G.); 2 Sports Drug Rehabilitation Center of Physical Education, Southwest University, Chongqing 400715, China; yangyi99@email.swu.edu.cn; 3 Key Lab of Exercise Training and Monitoring, Wuhan Sports University, Wuhan 430079, China; xuxia@whsu.edu.cn; 4 School of Sports Training, Wuhan Sports University, Wuhan 430079, China

**Keywords:** mental fatigue, boxing performance, time perceptual prediction, motion recognition

## Abstract

Objective: This study investigated the impact of mental fatigue on the temporal perceptual prediction of action recognition among boxers of different skill levels. Methods: A mixed experimental design of two (groups: Mental Fatigue Expert Group and Mental Fatigue Novice Group) × two (technique types: attack and defense) × three (time shields: −80 ms, −40 ms, and action start) was implemented. Twenty expert and novice boxers participated in this study. Mental fatigue was induced using a 45-min Stroop paradigm, and the effects were assessed using the VAS, Brog-20, BRUMS-F, and BRUMS-V. The experimental procedure for time perception was developed using E-prime 3.0, incorporating 36 videos depicting various attack and defense techniques, and reaction time and accuracy were recorded. Results: (1) A significant main effect on reaction time (RT) was observed (F (1,38) = 5.97, *p* < 0.05, η^2^ = 0.14) but not on accuracy (ACC), suggesting a pronounced influence of mental fatigue on novice boxers’ temporal perceptual prediction in action recognition; (2) significant main effects of skill types were noted in both RT (F (1,38) = 9.03, *p* < 0.05, η^2^ = 0.19) and ACC (F (1,38) = 18.496, *p* < 0.05, η^2^ = 0.327), indicating disparities in the recognition of offensive and defensive skills under mental fatigue; (3) temporal shielding significantly influenced both RT (F (2,76) = 31.42, *p* < 0.05, η^2^ = 0.45) and ACC (F (2,76) = 125.727, *p* < 0.05, η^2^ = 0.768), with −80 ms showing a lower RT and ACC compared to −40 ms and action initiation; (4) second-order interaction effects were present in both RT (F (2,76) = 9.85, *p* < 0.05, η^2^ = 0.21) and ACC (F (2,76) = 8.773, *p* < 0.05, η^2^ = 0.188), with the RT interaction suggesting a negative impact on perceptual prediction at −80 ms and a faster offensive RT than defensive RT. The ACC interaction indicated that under mental fatigue, −40 ms approached and exceeded −80 ms in both offensive and defensive actions, with higher ACC in offense than defense; and (5) a third-order interaction effect among group, technique type, and time shielding on RT (F (2,76) = 3.92, *p* < 0.05, η^2^ = 0.09) suggests that mental fatigue more significantly affects novice defensive technique RT than offensive technique. Conclusions: (1) The 45-min Stroop task effectively induced mental fatigue. (2) Mental fatigue negatively impacts both expert and novice boxers, with a more pronounced effect on experts’ defensive skills. (3) The −40 ms time perception is crucial for predicting action recognition as it approaches action initiation.

## 1. Introduction

Boxing demands high temporal and spatial perception from athletes, as they are required to make swift judgments and decisions in the field to capitalize on the best attack opportunities [1]. This process involves the perception, identification, and analysis of relevant environmental information, followed by the selection of appropriate responses by athletes [2]. Perception prediction, which utilizes partial or prior information to anticipate unknown events and facilitate information processing, plays a crucial role in this process and is vital for athletes in open sports [3]. Therefore, perceptual prediction in sports can be understood as the process where expert athletes selectively attend to more relevant cues to limit the amount of information processed, thereby generating a perceptual representation. This is essentially a process of making predictions based on limited cues [4]. These include action recognition, visual searches, and predictive processes [5]. Time and spatial perception predictions are specialized perceptions in boxing, in which athletes must coordinate their movements in both dimensions to optimize their athletic performance [6]. In boxing, it is not only crucial to emphasize strength and skill but also to possess accurate movement recognition to quickly identify an opponent’s technical moves and tactical intentions during a match. Action recognition is closely related to perceptual prediction. Action recognition can provide important informational support for perceptual prediction, helping athletes more accurately predict their opponents’ movements and the outcomes of competitions, thereby enhancing their performance [7]. Both offensive and defensive decisions are critical in boxing; effective offensive techniques, such as straight punches, swing punches, or hook punches, can score points or even knock down an opponent, while effective defensive techniques, such as dodging, blocking, or sliding, can help athletes avoid the opponent’s scoring and provide opportunities for effective counterattacks [8].

Mental fatigue is a psychobiological state resulting from prolonged high-intensity cognitive activity [9]. This state is characterized by exhaustion and discomfort due to mental stress and emotional strain [10]. According to the resource depletion theory, mental fatigue arises from the exhaustion of available cognitive resources due to the continuous allocation of attention [11]. It not only induces subjective feelings of fatigue and lack of energy but also adversely affects an individual’s physical and mental health, including emotional, cognitive, motivational, and physiological aspects [10]. In sports, mental fatigue can impair cognitive performance [12], such as perceptual prediction abilities, thereby negatively impacting athletic performance [13]. Specifically, studies have shown that mental fatigue can lead to significant declines in perceptual prediction, which is crucial for high-performance athletes [11]. Particularly after high-intensity training and competition, athletes undergo significant psychological preparation and adjustment, increasing the burden on their mental resources [14]. Consequently, mental fatigue may affect an individual’s neurobiological state, thereby influencing perceptual prediction abilities, including temporal and spatial perception prediction abilities. Time perception prediction, defined in conjunction with previous research [15], involves the use of incomplete sensory information to infer and predict the duration and sequence of objective events in sports. Time masking can be used to predict time perception [16]. This technique involves blocking video materials at different time points to form an observation period and obtain a progressive series, revealing perceptual prediction information during key time periods. The selection of temporal shielding points (−80 ms, −40 ms, and action initiation) was based on empirical evidence from perceptual prediction studies in combat sports. Specifically, −80 ms represents the early preparatory phase of an opponent’s action, allowing for the assessment of anticipatory skills, while −40 ms captures the critical window for rapid decision-making just prior to movement execution [4]. These time points align with the temporal dynamics of boxing, where split-second adjustments are required to predict and counter offensive and defensive maneuvers. According to a previous study [17], in terms of time shielding, −80 ms and −40 ms can demonstrate differences between expert athletes and novices. In the context of boxing, previous research has highlighted the importance of accurate movement recognition for effective offensive and defensive actions [1]. However, the specific impact of mental fatigue on the temporal perceptual prediction of action recognition in boxing remains less explored.

Despite these findings, several gaps remain in the literature. First, most existing studies have focused on the effects of mental fatigue on general cognitive performance, with limited research specifically addressing its impact on time perception prediction in sports like boxing [18,19]. Second, the differences in the effects of mental fatigue on offensive and defensive techniques have not been fully investigated [20,21,22]. Third, the critical time points for action recognition under mental fatigue conditions have not been clearly identified [23]. Finally, the interaction between skill level and mental fatigue in the context of temporal perceptual prediction has not been adequately explored [24].

To address these gaps, the present study aims to examine the impact of mental fatigue on the temporal perceptual prediction of action recognition among boxers of different skill levels. Specifically, we investigate whether mental fatigue differentially affects the recognition of offensive and defensive techniques and identify the critical time points for action recognition under mental fatigue conditions. Based on the literature and the nature of boxing performance, we hypothesize that (1) mental fatigue will significantly impair temporal perceptual prediction, primarily by increasing reaction time (RT) rather than reducing accuracy (ACC); (2) novice boxers will exhibit greater vulnerability to mental fatigue compared to experts, especially in defensive techniques; (3) offensive techniques will maintain relatively stable temporal perceptual prediction under mental fatigue, whereas defensive techniques will be more affected; and (4) the −40 ms time shield point will be critical for action recognition, as it provides sufficient information for accurate prediction, unlike the −80 ms point. This study will provide valuable insights into how mental fatigue influences boxing performance and inform future training and competition strategies.

## 2. Subjects and Methods

### 2.1. Subjects

Participants were recruited from among 20 individuals (16 males and 4 females) from a sports training major at a renowned sports university, all of whom were national first-class elite boxers.

The participants’ characteristics included the average age (20.30 ± 1.66 years), average height (1.76 ± 6.47 m), average body weight (72.43 ± 9.91 kg), and average training duration (6.58 ± 2.71 years). Additionally, a novice group comprising 20 male boxing beginners was recruited with an average age of (21.45 ± 1.99 years), average height of (1.75 ± 6.52 m), and an average weight of (73.89 ± 10.82 kg). Participants were recruited from among 20 individuals (16 males and 4 females) from a sports training major at a renowned sports university, all of whom were national first-class elite boxers.

All the participants exhibited normal intelligence and physical health, had no history of mental illness, and had either normal or corrected-to-normal vision. They had not previously engaged in any perceptual prediction exercise or received related guidance. Each participant voluntarily consented to participate in this study; abstained from alcohol, caffeine, and sleep deprivation within 24 h before this experiment; and received a gift upon completion. The study protocol was approved by the Ethics Committee of the School of Sports Medicine at Wuhan Sports University (approval number: 2024013).

### 2.2. Experiment Design

The experimental design employed a mixed-factorial approach with three factors: 2 (groups: Mental Fatigue Expert Group and Mental Fatigue Novice Group) × 2 (technique types: attack and defense) × 3 (time blocking: −80 ms, −40 ms, and action initiation). The group factor was a between-subjects factor, whereas technique type and time blocking were within-subject factors. The dependent variables were accuracy and reaction time for predicting motion decisions based on temporal perception.

### 2.3. Experiment Material

Mental fatigue was induced using the Stroop paradigm [19,25], which has been established to be more stable over a 45-min duration compared to a 30-min induction [26]. The Stroop task was programmed using E-prime 3.0 (Psychology Software Tools, Inc., Pittsburgh, PA, USA) software and initiated with the following instructions:

Thank you for this test. Please maintain your focus at the center of the screen throughout the test. A ‘+’ fixation point appears, followed by a colored word. Your task is to determine whether the font color matches the meaning of the word. If they match, press the ‘F’ key; if they do not, press the ‘J’ key. Respond as quickly and accurately as possible with feedback provided after each response. Failure to respond within 1.5 s or incorrect responses were recorded as errors. The test calculates accuracy and reaction time, with the top three performers receiving additional rewards. Press any key to begin.

After instructions, a 500 ms fixation point ‘+’ was presented, followed by a 1500 ms stimulus. Participants need to respond to the stimulus by pressing a button, and there will be 500 ms feedback after each stimulus. The Stroop paradigm consisted of three blocks, each with 300 traits lasting 15 min, for a total of 900 traits lasting 45 min.

The materials for the motion decision-making task based on time perception prediction were filmed using an iPhone 12 from the perspective of the subjects, with three boxers invited to a training center for video recording. The phone was positioned approximately 0.5 m above the ground and 2 m away from the athletes. Athletes were instructed to face the camera, assume a ready stance, and perform in-place technical movements for both attack and defense. Offensive actions included straight punches, hook punches, and swinging punches, and defensive actions included arm hugging, dodging, and blocking. Each movement was performed twice within 2 s. The videos were edited using Adobe Premier (Adobe Inc., San Jose, CA, USA), resulting in 99 videos (54 offensive and 45 defensive) based on the three blocking conditions. After evaluation by a coach and three graduate boxing students using a five-point rating scale, 77 videos were deemed suitable for experimental testing. The experimental program, developed using E-prime 3.0, included 36 formal experimental videos and 4 practice videos. Inter-rater reliability was ensured through a five-point rating scale used by the coach and three graduate boxing students to evaluate the videos, ensuring the final selection of 77 suitable videos for experimental testing.

Due to the negative impact of mental fatigue on subjective evaluations, several subjective rating scales have been validated as effective tools for assessing the state of mental fatigue. These include the Brunel Mood Scale (BRUMS-C, V, and F), the Visual Analog Scale (VAS), and the Borg-20 rating scale of perceived exertion (Borg-20) [11]. The following scales were used to assess the state of mental fatigue: The Visual Analog Scale (VAS) has demonstrated high reliability in mental fatigue research, with a test–retest reliability (ICC) of 0.69 (95% CI: 0.53–0.81) [27]. The Chinese version of the Brunel Mood Scale (BRUMS-C) has shown good reliability and validity in cross-cultural validation, with internal consistency (Cronbach’s α) ranging from 0.77 (fatigue) to 0.87 (anger) across its subscales. Confirmatory factor analysis (CFA) confirmed its structural validity (CFI ≥ 0.90, RMSEA ≤ 0.06), indicating its suitability for assessing mood states in Chinese academic settings [28]. The Borg-20 scale, widely used and validated, demonstrates high validity in assessing subjective fatigue [29] Table S1.

### 2.4. Experiment Process

This experiment was divided into two sessions: practice and formal. The practice session consisted of 4 trials, while the formal session comprised 36 trials, with 18 offensive and 18 defensive techniques. The experimental duration was approximately 3–4 min to closely simulate the duration of an actual boxing round.

**Figure 1:**
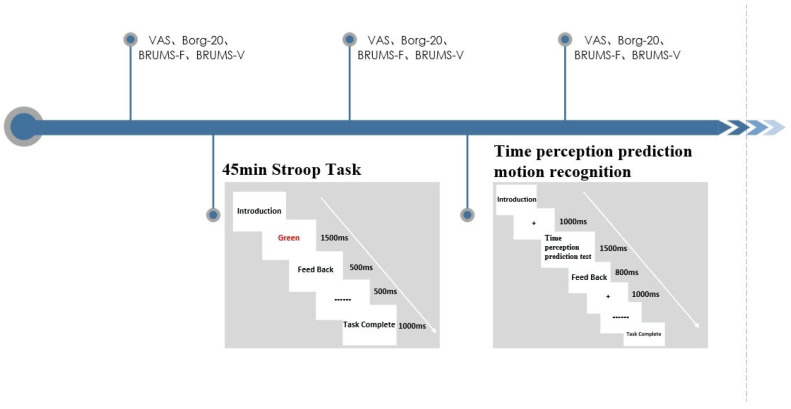
Experimental process of time perception prediction motion recognition.

The participants were invited to the laboratory for testing. Upon arrival, they signed an informed consent form, were assigned a code, and provided their demographic information (name, sex, age, major, grade, height, weight, group, and training years). Height and weight were measured onsite. Participants completed the Chinese versions of the Brunel Mood Scale (BRUMS-C), Visual Analog Scale (VAS), and Borg-20 rating scale of perceived exertion (Borg-20) before commencing formal testing. They then completed a 45-min Stroop paradigm to induce mental fatigue. After the task, BRUMS-C, VAS, and Brog-20 measurements were taken again, followed by the perceptual prediction task for motion decision-making. Videos of the boxing techniques were played randomly on a computer screen, and the participants were required to judge the techniques based on the observed content. For ‘straight punching’, press 1; for ‘hook punching’, press 2; for ‘swing punching’, press 3; for ‘arm hugging’, press 4; for ‘dodging’, press 5; and for ‘blocking’, press 6. The participants were required to respond within a given timeframe, with failure to respond to the considered errors. A practice session consisting of four trials was conducted before the formal experiment to familiarize the participants with the process. Final measurements of the BRUMS-C, VAS, and Borg-20 were performed upon completion (Figure 1). In subsequent expressions, 1, 2, and 3, respectively, refer to the subjective evaluation scores at three different time points.

### 2.5. Statics and Analysis

The experimental results mainly include descriptive statistics of RT and ACC in the Stroop paradigm, BRUMS-C scale only recording two subdimensions of BRUMS-F (fatigue) and BRUMS-V (vitality), one-way ANOVA of VAS and Borg-20, descriptive statistics of time perception prediction for action recognition and motion decision-making, and three-factor repeated measures ANOVA. SPSS 26.0 software was used for data analysis and processing, and Graphpad Prism 8 was used for plotting.

The experimental results primarily included descriptive statistics of reaction time (RT) and accuracy (ACC) in the Stroop paradigm, BRUMS-C scale recordings of the BRUMS-F (fatigue) and BRUMS-V (vitality) subdimensions, one-way ANOVA of VAS and Borg-20, descriptive statistics of time perception prediction for action recognition and motion decision making, and three-factor repeated measures ANOVA. Before conducting the repeated measures ANOVA, we tested the assumptions of normality and sphericity. Normality was assessed using the Shapiro–Wilk test, and sphericity was tested using Mauchly’s test. In case of violations of sphericity, we applied the Greenhouse–Geisser correction. Additionally, homogeneity of variance was tested using Box’s test. To provide a comprehensive understanding of the effect sizes reported in our study, we have included the interpretation thresholds for the partial eta-squared (η^2^) values. According to commonly accepted guidelines, η^2^ values can be interpreted as follows: a small effect size (η^2^ = 0.01) indicates a weak relationship between variables, which may not be very significant in practical applications; a medium effect size (η^2^ = 0.06) indicates a moderate relationship between variables, which is usually detectable through data analysis; and large effect size (η^2^ ≥ 0.14) indicates a strong relationship between variables, which is typically very evident and has practical significance [30]. Data analysis and processing were conducted using SPSS software (version 26.0), and GraphPad Prism 8 (GraphPad Software Inc., San Diego, CA, USA) was used for data visualization.

## 3. Results

The study findings are presented herein, encompassing data on mental fatigue induction, Visual Analog Scale (VAS), and Borg-20 scale scores, and an analysis of response times and accuracy rates in time perception prediction.

### 3.1. Mental Fatigue (MF)

The induction of mental fatigue was induced using the Stroop paradigm for 45 min. The mean and standard deviation of RT and ACC in the Stroop paradigm for the MF boxing expert group and MF boxing novice group are described statistically, as shown in Table 1.

### 3.2. VAS, Borg-20, BRUMS-F, and BRUMS-V Scale Scores

The VAS scores revealed a significant difference between the MF expert and novice groups (F (1,18) = 8.36, *p* = 0.005 < 0.05), with the novice group exhibiting higher scores than the expert group. Within the expert group, a significant increase was observed from VAS1 to VAS2 (*p* < 0.05) and from VAS1 to VAS3 (*p* < 0.05), with no significant difference between VAS2 and VAS3 (*p* > 0.05). The findings of the novice group mirrored these findings. This indicates that both groups experienced a significant increase in VAS scores after the Stroop task compared to pre-task levels.

Borg-20 scale scores showed no significant differences between the expert and novice groups (F (1,18) = 2.37, *p* = 0.126 > 0.05). However, in the expert group, a significant increase was noted from Brog-20 scale 1 to Borg-20 scale 2 (*p* < 0.05) and from Borg-20 scale 1 to Borg-20 scale 3 (*p* < 0.05), with no significant difference between Borg-20 scale 2 and Borg-20 scale 3 (*p* > 0.05). The novice group exhibits a similar trend. These results suggest that both groups had significantly elevated Borg-20 scale scores following the Stroop task compared with pre-task levels.

**Figure 2:**
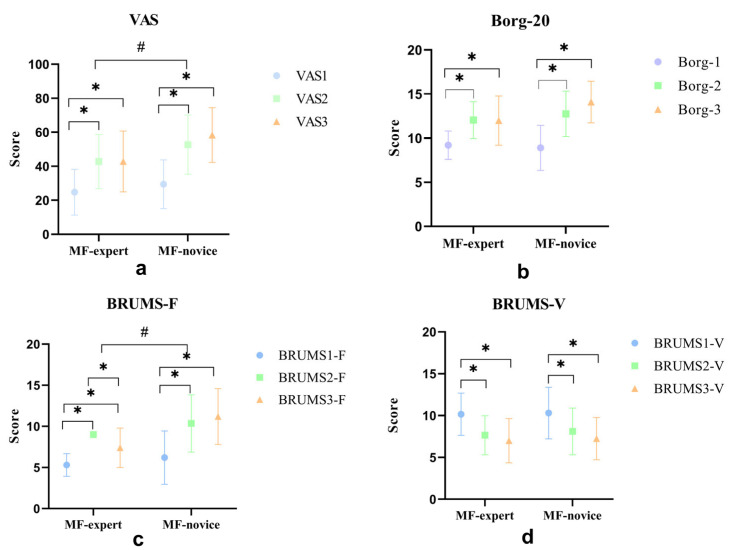
VAS, Borg-20, BRUMS-F, and BRUMS-V scale scores for MF expert group and MF novice group. * indicates a significance level of 0.05. “#” indicates that there is a significant difference between the MF-expert group and the MF-novice group. (**a**) VAS scores of expert and novice groups at three time points in mental fatigue; (**b**) Borg-20 scores of expert and novice groups at three time points in mental fatigue; (**c**) BRUMS-F scores of expert and novice groups at three time points in mental fatigue; and (**d**) BRUMS-V scores of expert and novice groups at three time points in mental fatigue.

The VAS, Borg-20, and BRUMS-F scores collectively indicated an increase in subjective fatigue after the Stroop task. Conversely, the BRUMS-V vitality dimension scores decreased after the task, indicating reduced vitality (Figure 2).

### 3.3. Reaction Time (RT)

Descriptive statistics for RT in the time perception prediction and motion decision-making experiments are presented in Table 2. Inferential statistical analysis was conducted using repeated-measures analysis of variance (ANOVA) within subjects. Box’s test for homogeneity of variance indicated variance homogeneity across groups (*p* = 0.376 > 0.05). Multivariate ANOVA revealed significant main effects of the technique type (*p* = 0.005, *p* < 0.01), whereas the interaction between the technique type and group was not significant (*p* = 0.087, *p* > 0.05). The main effect of shielding time was significant (*p* = 0.000, *p* < 0.01), as was the interaction between technique type and shielding time (*p* = 0.001, *p* < 0.01), and the three-way interaction involving group, technique type, and shielding time (*p* = 0.031, *p* < 0.05). The main effect of the group was significant (*p* = 0.019, *p* < 0.05). Sphericity assumptions were not violated by technique type, and the time-shielding effects were consistent with the sphericity assumption (*p* = 0.156, *p* > 0.05). The main effect of the group was significant, with F (1,38) = 5.97, *p* < 0.05, η^2^ = 0.14. The main effect of technique type was significant, with F (1,38) = 9.03, *p* < 0.05, η^2^ = 0.19. The interaction between group and technique type was not significant, with F (1,38) = 3.10, *p* > 0.05, η^2^ = 0.075. The main effect of shielding time was significant, F (2,76) = 31.42, *p* < 0.05, η^2^ = 0.45. The interaction between group and time shielding was not significant, with F (2,76) = 0.18, η^2^ = 0.01; the interaction between technique type and time shielding was significant, with F (2,76) = 9.85, *p* < 0.05, η^2^ = 0.21; and the three-way interaction was significant, with F (2,76) = 3.92, *p* < 0.05, η^2^ = 0.09. The results of the multivariate ANOVA were consistent with those of the univariate ANOVA.

Multiple comparisons were conducted due to the significant main effect of time shielding and its three levels. The interaction between technique type and shielding time was significant, necessitating simple effects tests. The three-way interaction involving group, technique type, and time shielding was also significant and requires further investigation.

Multiple comparison results for time shielding revealed a significant difference between level 1 (−80 ms) and level 3 (action initiation) of factor C, indicating that the RT at −80 ms was shorter than that at action initiation (*p* = 0.005, *p* < 0.05). There was also a significant difference between level 1 (−80 ms) and level 2 (−40 ms), indicating that the RT was shorter at −80 ms compared to −40 ms. No significant difference was observed between level 2 (−40 ms) and level 3 (action initiation) (*p* = 0.421, *p* > 0.05).

Simple effects tests for the interaction of technique type and time shielding (B × C) indicated no significant difference between −80 ms and −40 ms time shields under offensive conditions. However, the difference between −80 ms time shielding and action initiation was significant (*p* < 0.05), with action initiation RT being higher than −80 ms. Similarly, there was a significant difference between −40 ms and action initiation, with −40 ms shielding time being faster than action initiation. Under defensive conditions, a significant difference in time shielding was observed between −80 ms and −40 ms (*p* < 0.05), with RT being faster at −40 ms than at −80 ms. No significant difference was found between −80 ms and action initiation, while a significant difference was observed between −40 ms and action initiation (*p* < 0.05), with −40 ms being faster than action initiation.

In terms of time blocking, a significant difference in offensive and defensive techniques was observed at −80 ms (*p* < 0.05), with no significant difference at −40 ms (*p* > 0.05). Under the action initiation conditions, no significant differences in offensive and defensive techniques were found (*p* > 0.05). This suggests that there is a difference in RT between offensive and defensive techniques at −80 ms, with offensive techniques having shorter action recognition times.

The simple effects test results showed no significant difference between the MF expert and novice groups at −40 ms (*p* = 0.44, *p* > 0.05). At −80 ms, no significant difference was observed (*p* = 0.08, *p* > 0.05). At action initiation, a marginally significant difference was observed (*p* = 0.06). Under defensive conditions, a significant difference was found between the MF expert and novice groups at −40 ms (*p* = 0.005, *p* < 0.05). At −80 ms, no significant difference was observed (*p* = 0.20, *p* > 0.05). At action initiation, a marginally significant difference was observed (*p* = 0.06). The MF expert group may exhibit a shorter perceived reaction time than the MF novice group when initiating both offensive and defensive actions. Additionally, the MF expert group may recognize defensive actions earlier than the MF novice group at −40 ms.

**Figure 3:**
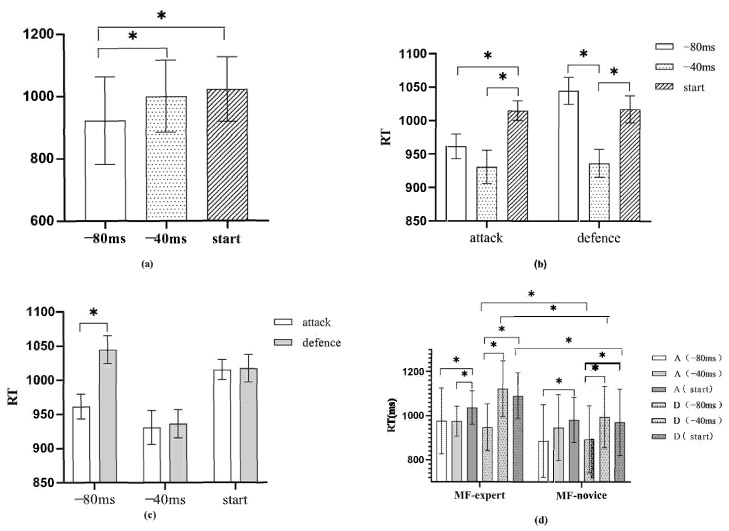
(**a**) is the main effect of time shielding, (**b**,**c**) is the second-order interaction effect (RT) between technology type and time shielding, and (**d**) is the third-order interaction effect (RT) between group, technology type, and time shielding. * indicates a significance level of 0.05.

Under the MF expert group conditions, significant differences in attack and defense technical types were observed at −40 ms (*p* = 0.00, *p* < 0.05), with no significant differences at −80 ms (*p* = 0.27, *p* > 0.05). At action initiation, significant differences in attack and defense technical types were observed (*p* = 0.04, *p* < 0.05). Under the MF novice group conditions, no significant differences in attack and defense technical types were observed at −40 ms and similarly at −80 ms (*p* = 0.77, *p* > 0.05). No significant differences were observed in action initiation (*p* = 0.68, *p* > 0.05). The MF expert group demonstrated a shorter reaction time to defensive movements and faster recognition of whether the opponent intended to attack or defend under −40 ms and action initiation conditions, whereas no such difference was observed in the MF novice group. This suggests that the MF novice group had weaker recognition of attack and defense technical types compared to the MF expert group under the three blocking conditions. Under the MF expert group conditions, no significant difference was observed in time blocking between −40 ms and −80 ms when the technical type was offensive (*p* = 0.98, *p* > 0.05), and a significant difference was found in time blocking between −40 ms and −80 ms when the technical type was defensive (*p* = 0.012, *p* < 0.05). No significant difference was observed in time blocking between −40 ms and −80 ms when the technical type was defensive, and no significant difference was observed in time blocking between −40 ms and −80 ms when the technical type was offensive. A significant difference was observed in time blocking between −40 ms and −80 ms when the technical type was offensive in the MF novice group (*p* = 0.004, *p* < 0.05). The difference between −40 ms and action initiation was not significant (*p* = 0.28, *p* > 0.05), while the difference between −80 ms and action initiation time shielding was significant (*p* = 0.004, *p* < 0.05); when the technical type was defense, there was a significant difference in time blocking between −40 ms and −80 ms (*p* = 0.002, *p* < 0.05), but there was no significant difference in time blocking between −40 ms and action initiation (*p* = 0.98, *p* > 0.05), and there was a significant difference in time blocking between −80 ms and action initiation (*p* = 0.007, *p* < 0.05). This indicates that in the MF expert group, the time perception judgment of offensive techniques is faster at −40 ms than at action initiation, while the time perception judgment of defensive techniques is faster at −40 ms than at −80 ms, and the time perception of action initiation is also faster at −80 ms. The MF novice group showed slower time perception in action initiation for offensive techniques compared to −80 ms, while defensive techniques had longer time perception reaction times for −40 ms compared to −80 ms and longer time perception reaction times for −80 ms compared to action initiation (Figure 3).

### 3.4. Accuracy (ACC)

Descriptive statistics for the ACC in the motion decision experiment based on time perception prediction are detailed in Table 3. Inferential statistical analyses were performed using repeated-measures measures ANOVA of variance. Box’s test for homogeneity of variance indicated variance heterogeneity across groups (*p* = 0.042, *p* < 0.05), prompting the use of the Greenhouse–Geisser correction. Multivariate ANOVA revealed significant main effects of the technique type (*p* = 0.000, *p* < 0.01), whereas the interaction between the technique type and group was not significant (*p* = 0.413, *p* > 0.05). The main effect of time shielding was significant (*p* = 0.000, *p* < 0.01), as was the interaction between technique type and time shielding (*p* = 0.004, *p* < 0.01) but not the interaction between group and time shielding (*p* = 0.467, *p* > 0.05) or the three-way interaction (*p* = 0.360, *p* > 0.05). The main effect of the group was not significant (*p* = 0.482). Sphericity assumptions were not violated by technique type, and the time-shielding effects were consistent with the sphericity assumption (*p* = 0.526, *p* > 0.05). The main effect of the group was not significant, with F (1,38) = 0.898, *p* < 0.05, η^2^ = 0.023. The main effect of technique type was significant, with F (1,38) = 18.496, *p* < 0.05, η^2^ = 0.327. The interaction between group and technique type was not significant, with F (1,38) = 0.684, *p* > 0.05, η^2^ = 0.018. The main effect of time shielding was significant, with F (2,76) = 125.727, *p* < 0.05, η^2^ = 0.768. The interaction between group and time shielding was not significant, F (2,76) = 0.937, η^2^ = 0.024; the interaction between technique type and time shielding was significant, F (2,76) = 8.773, *p* < 0.05, η^2^ = 0.188. The three-way interaction was not significant with F (2,76) = 1.018, *p* < 0.05, η^2^ = 0.026. The results of the multivariate ANOVA were consistent with those of the univariate ANOVA.

Multiple comparisons were conducted due to the significant main effect of time shielding and its three levels. The interaction between technique type and shielding time was significant, necessitating simple effects tests.

Multiple comparison results for time shielding revealed a significant difference between level 1 (−80 ms) and level 2 (−40 ms) of factor C, with ACC at −40 ms being higher than ACC at −80 ms (*p* = 0.000, *p* < 0.05). There was also a significant difference between levels 1 (−80 ms) and 3 (action initiation), with the ACC at action initiation being higher than that at −80 ms (*p* = 0.000, *p* < 0.05). No significant difference was observed between levels 2 and 3, indicating no difference between the ACC at −80 ms and the ACC at action initiation (*p* = 0.380, *p* > 0.05).

**Figure 4:**
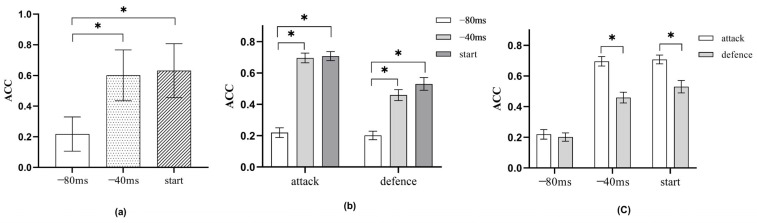
(**a**) is the main effect of time shielding, and (**b**,**c**) is the second-order interaction effect (ACC) between technology type and time shielding. * indicates a significance level of 0.05.

The results of the BC simple effects test showed that in terms of technical type (B), there was a significant difference (*p* > 0.05) between the −40 ms time shield and the −80 ms time shield under the attack (B1) condition, with ACC under the attack condition being higher at −40 ms than at −80 ms. There was no significant difference between 40 ms and action initiation (*p* > 0.05). There was a significant difference (*p* < 0.05) between the 80 ms time shield and action initiation, with the ACC of action initiation under offensive conditions being higher than −80 ms. Under defensive conditions (B2), there was a significant difference (*p* < 0.05) between the −40 ms time shield and the −80 ms time shield, with the ACC of the −40 ms time shield being higher than that of the −80 ms time shield. There was no significant difference (*p* > 0.05) between the 40 ms shielding time and action initiation. The difference between 80 ms and action initiation was significant (*p* < 0.05), with ACC at action initiation being higher than that at −80 ms time masking. In terms of time shielding (C), under the condition of −40 ms (C1), there was a significant difference in the technical types of attack and defense (*p* < 0.05), with the ACC of attack being higher than that of defense. Under the condition of −80 ms (C2), there was no significant difference in the technical types of attack and defense (*p* > 0.05). However, under the condition of action initiation (C3), there was also a significant difference in the technical types of attack and defense (*p* < 0.05), with the ACC of attack being higher than that of defense (Figure 4).

## 4. Discussion

This study was designed to investigate the effects of mental fatigue on the temporal perceptual prediction of action recognition among boxers with varying skill levels. Utilizing the Stroop paradigm for 45 min, we successfully induced mental fatigue and subsequently assessed the recognition of offensive and defensive technical movements by expert and novice boxers at three distinct time-blocking points [2,19,25].

Our findings indicate that the 45-min Stroop task effectively induced mental fatigue, as evidenced by significant changes in subjective fatigue and vitality indicators, such as the VAS, Brog-20, and BRUMS-F, before and after the task. This aligns with previous research suggesting that prolonged cognitive tasks can lead to mental fatigue, affecting cognitive performance, including perceptual prediction [9,12,13].

The main effect of the group on reaction time (RT) was significant, but not on accuracy (ACC), suggesting that while there was a significant difference in reaction time between expert and novice boxers, accuracy remained unaffected. This discrepancy may be attributed to the expert group’s long-term specialized training, which confers an advantage in RT but not necessarily in ACC, potentially due to the impact of mental fatigue on decision-making accuracy [31,32]. The current study also revealed a dissociation between reaction time and accuracy. This dissociation is believed to be caused by mental fatigue resulting from prolonged cognitive tasks, which leads to reduced activity in brain regions associated with control [33]. Consequently, athletes adjust their strategies during the information integration phase, extending processing time to maintain basic decision-making accuracy.

Regarding technique type, both the RT and ACC exhibited main effects, highlighting the differences in the recognition of offensive and defensive techniques during mental fatigue. Our results suggest that offensive techniques are recognized more quickly and accurately than defensive techniques, which is consistent with previous research indicating that cognitive fatigue has a greater impact on offensive actions [12,22]. Compared to defense, offense may activate more primitive action patterns (such as automated processing dominated by the basal ganglia), while defense involves more complex cognitive monitoring (such as conflict detection by the dorsolateral prefrontal cortex). This suggests that mental fatigue may have a greater impact on automated processing [34].

The main effect of time shielding was also significant for both RT and ACC, with the −80 ms time point showing lower RT and ACC compared to −40 ms and action initiation. This finding suggests that as action initiation approaches, the advantage of expert boxers diminishes, potentially because of the influence of mental fatigue on information utilization [23,35].

The significant second-order interaction effects between technique type and time shielding on both RT and ACC indicate that the −40 ms time shield point provides sufficient information for participants to recognize action patterns. Furthermore, the third-order interaction effects between group, technique type, and time shielding on RT suggest that mental fatigue has a more pronounced impact on novice defensive techniques than on offensive techniques [4,21]. In line with previous studies supporting the Cognitive Resource Depletion Model [36], it is suggested that time-specific attentional focus training be adopted under mentally fatigued conditions to optimize decision-making strategies. This approach will allow for a better understanding of how athletes utilize situational information in competitions and facilitate the development of effective training programs that can be transferred from training to competition [37].

Previous studies demonstrated that mental fatigue can affect the accuracy of passing decisions in ball sports and increase reaction times during decision-making. However, the impact of mental fatigue on decision-making in different sports varies, with the current research focusing primarily on ball sports such as football and basketball [19,20,38]. Our findings contribute to the understanding of how mental fatigue affects perceptual predictions in boxing, a domain that has been less explored in the literature.

To mitigate the adverse effects of mental fatigue (MF) on temporal prediction in sports training, targeted interventions can be strategically applied: Non-invasive neuromodulation techniques, such as anodal transcranial direct current stimulation (tDCS) targeting the dorsolateral prefrontal cortex (DLPFC), have been shown to enhance cognitive control under MF, with studies in cyclists and swimmers demonstrating preserved reaction time and decision accuracy during fatigue. This approach could be synergized with sport-specific drills (e.g., video simulations of opponent actions at −80/−40 ms time points) to optimize temporal anticipation in boxing. Acute caffeine supplementation (3–6 mg/kg) antagonizes adenosine receptors, sustaining alertness and dopamine-driven motivation, which is critical for rapid defensive adjustments (e.g., blocking at −40 ms) in combat sports. Mindfulness training, such as brief breath-focused meditation sessions, reduces task-irrelevant thoughts and improves attentional focus, as evidenced by volleyball players exhibiting faster decision-making post-intervention, a strategy transferable to boxing scenarios requiring split-second predictions. Additionally, olfactory interventions using neutral pleasant odors (e.g., citrus) activate reward-related brain regions, potentially resetting cognitive load during rest intervals and enhancing temporal anticipation in subsequent training rounds. These evidence-based strategies, tailored to sport-specific demands, offer practical solutions to counteract MF-induced declines in temporal prediction, though further validation in combat sports contexts is needed to refine implementation protocols [11].

In conclusion, the current study provides evidence that mental fatigue negatively affects both expert and novice boxers, with a more significant effect on defensive skills among experts. The −40 ms time perception is identified as a critical point for action recognition, and the effects of mental fatigue are not linear across various levels of expertise, types of techniques, and time-blocking points [18,39,40].

## 5. Conclusions

This study provided several key findings regarding the impact of mental fatigue on temporal perceptual prediction in boxers with varying skill levels. In this section, we summarize our conclusions and implications.

First of all, the 45-min Stroop task effectively induced mental fatigue, as evidenced by significant changes in subjective fatigue and vitality indicators such as the VAS, Brog-20, and BRUMS-F scores before and after the task. In the second place, in a state of mental fatigue, the reaction time (RT) and accuracy (ACC) of the offensive techniques were higher than those of the defensive techniques. This suggests that mental fatigue may disproportionately affect the execution and recognition of defensive actions in boxing. Furthermore, the extraction of information at −40 ms was identified as a key time point for action recognition compared to −80 ms and action initiation. This indicates that the temporal window of −40 ms may be crucial for predicting action recognition in boxing. Lastly, mental fatigue can have negative effects on experts and beginners, offense and defense, and different time-blocking points; however, this effect is not linear. This finding underscores the complexity of how mental fatigue influences boxing performance and suggests that the impact may vary depending on the specific context and individual characteristics.

These conclusions contribute to the growing body of literature on the effects of mental fatigue in sports, particularly in high-intensity and high-skill sports, such as boxing. Future research should explore the nuances of mental fatigue and its interaction with various performance metrics to develop targeted interventions for athletes.

## 6. Limitation and Future Research Outlook

While the current study provides valuable insights into the effects of mental fatigue on boxing performance, further research is warranted to explore the intricate relationships among mental fatigue, perceptual prediction, and decision-making in sports. The sample size in the current study was determined based on the researchers’ prior experience; a more rigorous approach would involve calculating an optimal sample size using G-power. Second, while the current study focused on temporal perception prediction under mental fatigue, future research could include a control group without mental fatigue to investigate differences in temporal perception prediction between states with and without mental fatigue. Another notable limitation is the gender imbalance in our participant sample, with only four female experts compared to 16 male experts and 20 male novices. This imbalance is due to the relatively low number of female participants in boxing, a sport traditionally dominated by males. Future research should aim to recruit a more balanced gender distribution to better understand the effects of mental fatigue across different genders. However, the limitation of our study is that while we reported effect sizes for the reaction time and accuracy data, we did not include effect sizes for the questionnaire data (VAS, BRUMS, and Brog-20). Future research should consider including effect sizes for all statistical analyses to provide a more comprehensive understanding of the results. While our results indicate that −40 ms is a statistically significant point for temporal perceptual prediction, the magnitude of the interaction effects suggests that this should be interpreted with caution in practical contexts.

The findings of this study have significant potential implications for practice and sports training. Coaches can design more targeted training programs based on the study results, considering the athletes’ mental fatigue status and the characteristics of offensive and defensive techniques. For example, by optimizing training plans, the negative impact of fatigue on athletes’ decision-making abilities can be reduced; in defensive technique training, the ability to recognize key information (such as at −40 ms) can be enhanced; and in offensive technique training, the athletes’ rapid recognition of offensive cues can be utilized to improve decision-making efficiency.

Furthermore, future research on the topic of mental fatigue and perceptual prediction in athletes could benefit from the application of advanced technologies such as eye-tracking, ERPs (Event-Related Potentials), and fNIRS (functional Near-Infrared Spectroscopy). These technologies can provide deeper insights into the neural mechanisms underlying the effects of mental fatigue on athletes’ performance. Additionally, this research topic is not only relevant to the field of sports but also holds potential for interdisciplinary collaboration. Collaboration with experts in psychology, physiology, and artificial intelligence could lead to more comprehensive and effective solutions for managing mental fatigue in sports.

## Figures and Tables

**Table 1:** RT and ACC of MF boxing expert group and MF boxing novice group in Stroop task.

Group	Mean	SD
MF Boxing Expert (RT)	571.50	62.19
MF Boxing Novice (RT)	576.50	55.17
MF Boxing Expert (ACC)	97.02	1.61
MF Boxing Novice (ACC)	96.94	1.82

There were 20 subjects in both groups, N = 40.

**Table 2:** Descriptive statistics of RT for time perception prediction motion recognition (M ± SD).

	Attack			Defense		
	−80 ms	−40 ms	Start	−80 ms	−40 ms	Start
BE	744.51 ± 62.02	811.16 ± 67.51	856.69 ± 71.30	855.86 ± 71.19	729.44 ± 60.08	784.64 ± 65.37
BN	833.73 ± 74.78	914.81 ± 82.05	919.79 ± 82.49	849.69 ± 76.20	932.45 ± 83.63	830.57 ± 74.55

BE is the boxing expert group, and BN is the boxing novice group.

**Table 3:** Descriptive statistics of ACC for time perception prediction motion recognition (M ± SD).

	Attack			Defense		
	−80 ms	−40 ms	Start	−80 ms	−40 ms	Start
BE	20.90 ± 18.58	75.25 ± 18.11	75.15 ± 11.51	20.00 ± 15.93	45.75 ± 22.89	56.80 ± 28.80
BN	23.60 ± 20.49	64.30 ± 21.01	67.45 ± 21.51	20.45 ± 18.86	47.30 ± 20.62	53.35 ± 24.19

BE is the boxing expert group, and BN is the boxing novice group.

## Data Availability

The data underlying this study are not publicly available at this time, as they are part of an ongoing research effort and are currently being further analyzed and integrated into a larger research context. The data were derived from a doctoral dissertation that is in the process of finalization. We plan to make the data available upon the completion of the dissertation and after ensuring that all necessary ethical and privacy considerations have been addressed. We will update this statement with the relevant data access information once the data are ready for public release.

## Supplementary Materials

The following supporting information can be downloaded at: https://www.mdpi.com/article/10.3390/sports13050154/s1, Table S1:summary table listing.

## References

[1] Wu R., Yang Q., Cui W., Gao D., Luo Y., Wang D. (2024). Relationship between visual ability assessment and punch performance in competition in male amateur boxers. Front. Physiol..

[2] Baker J., Cote J., Abernethy B. (2003). Sport-Specific Practice and the Development of Expert Decision-Making in Team Ball Sports. J. Appl. Sport. Psychol..

[3] Fortes L.S., Nascimento-Júnior J.R.A., Mortatti A.L., Lima-Júnior D.R.A.A., Ferreira M.E.C. (2018). Effect of Dehydration on Passing Decision Making in Soccer Athletes. Res. Q. Exerc. Sport..

[4] Causer J., Smeeton N.J., Williams A.M. (2017). Expertise differences in anticipatory judgements during a temporally and spatially occluded task. PLoS ONE.

[5] Mann D.T., Williams A.M., Ward P., Janelle C.M. (2007). Perceptual-cognitive expertise in sport: A meta-analysis. J. Sport. Exerc. Psychol..

[6] Travassos B., Duarte R., Vilar L., Davids K., Araújo D. (2012). Practice task design in team sports: Representativeness enhanced by increasing opportunities for action. J. Sport. Sci..

[7] Unenaka S., Ikudome S., Mori S., Nakamoto H. (2018). Concurrent Imitative Movement During Action Observation Facilitates Accuracy of Outcome Prediction in Less-Skilled Performers. Front. Psychol..

[8] Fortes L.S., Gantois P., de Lima-Júnior D., Barbosa B.T., Ferreira M.E.C., Nakamura F.Y., Albuquerque M.R., Fonseca F.S. (2023). Playing videogames or using social media applications on smartphones causes mental fatigue and impairs decision-making performance in amateur boxers. Appl. Neuropsychol. Adult.

[9] Proost M., Habay J., De Wachter J., De Pauw K., Rattray B., Meeusen R., Roelands B., Van Cutsem J. (2022). How to Tackle Mental Fatigue: A Systematic Review of Potential Countermeasures and Their Underlying Mechanisms. Sports Med..

[10] Congyuxin Y.S.S.F. (2024). Mental Fatigue in Sports: Causes, Effects, and Coping Strategies—A Review Study. Int. J. Acad. Res. Progress. Educ. Dev..

[11] Wu C., Zhao Y., Yin F., Yi Y., Geng L., Xu X. (2024). Mental Fatigue and Sports Performance of Athletes: Theoretical Explanation, Influencing Factors, and Intervention Methods. Behav. Sci..

[12] Sun H., Soh K.G., Roslan S., Wazir M.R.W.N., Soh K.L. (2021). Does mental fatigue affect skilled performance in athletes? A systematic review. PLoS ONE.

[13] Chen X., Ji Z., Wang Y., Xu J., Wang L., Wang H. (2023). Bibliometric analysis of the effects of mental fatigue on athletic performance from 2001 to 2021. Front. Psychol..

[14] Lu C., Xu J. (2023). Influences of Physical, Environment, Task, Timing, Learning, Emotion, Perspective (PETTLEP) Intervention on Psychological Resilience, Psychological Skills, Anxiety and Depression of Athletes. Iran. J. Public. Health.

[15] Huang X., Song Q. (2004). Thinking about the Orical Model of Time Cognition. J. Southwest China Norm. Univ..

[16] Scharlau I. (2007). Temporal processes in prime–mask interaction: Assessing perceptual consequences of masked information. Adv. Cogn. Psychol..

[17] Hong-peng Z., Zhao-yang G. (2014). Predict Behavior and Neural Mechanism of Excellent Sanda Athletes Under Different Situations. J. Shenyang Sport. Univ..

[18] Smith M.R., Zeuwts L., Lenoir M., Hens N., De Jong L.M., Coutts A.J. (2016). Mental fatigue impairs soccer-specific decision-making skill. J. Sports Sci..

[19] Pageaux B., Lepers R. (2018). The effects of mental fatigue on sport-related performance. Prog. Brain Res..

[20] Fortes L.S., Lima-Junior D., Nascimento-Júnior J.R.A., Costa E.C., Matta M.O., Ferreira M.E.C. (2019). Effect of exposure time to smartphone apps on passing decision-making in male soccer athletes. Psychol. Sport. Exerc..

[21] Gantois P., Caputo F.M., Lima-Junior D., Nakamura F.Y., Batista G.R., Fonseca F.S., Fortes L.S. (2020). Effects of mental fatigue on passing decision-making performance in professional soccer athletes. Eur. J. Sport. Sci..

[22] Le Mansec Y., Pageaux B., Nordez A., Dorel S., Jubeau M. (2018). Mental fatigue alters the speed and the accuracy of the ball in table tennis. J. Sports Sci..

[23] Sun W.F., Wang B., Guo D., Zhang D.X., Lin R.H. (2021). Research on Processing Superiority of Advance Cues for Elite Sanda. Athl. J. Cap. Univ. Phys. Educ. Sports.

[24] Martin K., Staiano W., Menaspà P., Hennessey T., Marcora S., Keegan R., Thompson K.G., Martin D., Halson S., Rattray B. (2016). Superior Inhibitory Control and Resistance to Mental Fatigue in Professional Road Cyclists. PLoS ONE.

[25] Staiano W., Bosio A., Piazza G., Romagnoli M., Invernizzi P.L. (2019). Kayaking performance is altered in mentally fatigued young elite athletes. J. Sports Med. Phys. Fit..

[26] Yang W., Li J., Zhao S. (2024). Comparison of the effects of mental fatigue induction tasks. Chin. J. Tissue Eng. Res..

[27] Brietzke C., Vinícius Í., Franco-Alvarenga P.E., Canestri R., Goethel M.F., Santos L.E.R., Viana B., Santos T.M., Pires F.O. (2021). Proof-of-Concept and Test-Retest Reliability Study of Psychological and Physiological Variables of the Mental Fatigue Paradigm. Int. J. Env. Res. Pub He.

[28] Hasan M.M., Khan M.H.A. (2022). Bangla version of the Brunel Mood Scale (BRUMS): Validity, measurement invariance and normative data in non-clinical sample. Heliyon.

[29] Pancardo P., Hernández-Nolasco J.A., Acosta-Escalante F. (2018). A Fuzzy Logic-Based Personalized Method to Classify Perceived Exertion in Workplaces Using a Wearable Heart Rate Sensor. Mob. Inf. Syst..

[30] Fritz C.O., Morris P.E., Richler J.J. (2012). Effect size estimates: Current use, calculations, and interpretation. J. Exp. Psychol. Gen..

[31] Shu-Ming W., Jian-chen Z., Xiao-jian Y. (2009). A Research on Performance of Perceptual-motor Skill Training For Badminton Players. J. Beijing Sport. Univ..

[32] Smith M.R., Thompson C., Marcora S.M., Skorski S., Meyer T., Coutts A.J. (2018). Mental Fatigue and Soccer: Current Knowledge and Future Directions. Sports Med..

[33] Van Cutsem J., Van Schuerbeek P., Pattyn N., Raeymaekers H., De Mey J., Meeusen R., Roelands B. (2022). A drop in cognitive performance, whodunit? Subjective mental fatigue, brain deactivation or increased parasympathetic activity? It’s complicated!. Cortex.

[34] Nakamoto H., Mori S. (2012). Experts in fast-ball sports reduce anticipation timing cost by developing inhibitory control. Brain Cogn..

[35] Hong-peng Z., Cheng-lin Z. (2010). Features and Neural Mechanism of Feature Searching of Elite Sanda Athlete. J. Shanghai Univ. Sport..

[36] van der Linden D., Frese M., Meijman T.F. (2003). Mental fatigue and the control of cognitive processes: Effects on perseveration and planning. Acta Psychol..

[37] Williams A.M., Jackson R.C. (2019). Anticipation in sport: Fifty years on, what have we learned and what research still needs to be undertaken?. Psychol. Sport. Exerc..

[38] Fortes L.S., De Lima-Junior D., Fiorese L., Nascimento-Junior J., Mortatti A.L., Ferreira M. (2020). The effect of smartphones and playing video games on decision-making in soccer players: A crossover and randomised study. J. Sports Sci..

[39] Fortes L.S., Lima-Junior D., Barbosa B.T., Faro H.K.C., Ferreira M.E.C., Almeida S.S. (2022). Effect of mental fatigue on decision-making skill and visual search behaviour in basketball players: An experimental and randomised study. Int. J. Sport. Exerc. Psychol..

[40] Trecroci A., Boccolini G., Duca M., Formenti D., Alberti G. (2020). Mental fatigue impairs physical activity, technical and decision-making performance during small-sided games. PLoS ONE.

