# Supplementary material for: Mental Fatigue Impairs Temporal Perceptual Prediction: A Study on Boxing Performance Across Skill Levels
Source: Sports (Basel). 2025 May 20;13(5):154. doi: 10.3390/sports13050154 (PMC12115969; doi:10.3390/sports13050154)
Supplement: Supplementary file 1 [file sports-13-00154-s001.zip › sports-3600119-supplementary.pdf]

Table S1:summary table listing

Full Name	Abbreviation
Brunel Mood Scale	BRUMS
Visual Analog Scale	VAS
Borg-20 rating scale of perceived exertion	Borg-20

Explanation:

1. Brunel Mood Scale: The Brunel Mood Scale (BRUMS) is commonly used to assess emotional states. In the text, "BRUMS-C" refers to the Chinese version of the scale, while "BRUMS-V" and "BRUMS-F" refer to the vigor and fatigue dimensions of the scale, respectively.
2. Visual Analog Scale: The Visual Analog Scale (VAS) is often used to evaluate subjective feelings (such as pain or fatigue).
3. Borg-20 rating scale of perceived exertion: The Borg-20 scale is used to assess an individual's subjective perception of exertion.
